# Conditions and Pathways for Integrating Smart Care Robots Into Long-Term Care Facilities: Qualitative Study From Nursing Assistants’ Perspectives

**DOI:** 10.2196/97371

**Published:** 2026-10-21

**Authors:** Yawen An, Fengying Zhang, Yang Yang, Ruben Martin-Payo, Xiaofeng Xie

**Affiliations:** 1 West China School of Nursing / West China Hospital, Sichuan University, No. 24, South Section, First Ring Road, Chengdu, Sichuan, 610041, China, 86 15397611001; 2 School of Mathematics, Southwestern University of Finance and Economics, Chengdu, China; 3 SWUFE-UD Institute of Data Science, Southwestern University of Finance and Economics, Chengdu, China; 4 Department of Medicine, University of Oviedo, Oviedo, Spain; 5 Precam Research Group, Instituto de Investigación Sanitaria del Principado de Asturias, Oviedo, Spain

**Keywords:** smart care robots, long-term care facilities, nursing assistants, qualitative research, practical pathways

## Abstract

**Background:**

Smart care robots are increasingly viewed as a potential way to address care burden and support care quality in long-term care facilities. However, their integration into routine care practice remains limited, and the conditions required for their effective implementation are not yet well understood from the perspective of frontline nursing assistants.

**Objective:**

This study aimed to clarify nursing assistants’ core functional expectations of smart care robots in long-term care facilities and to explore the key conditions for their integration into practice.

**Methods:**

A qualitative descriptive study was conducted. Using purposive sampling, 31 frontline nursing assistants were recruited from 5 long-term care facilities in Chengdu, Sichuan Province, China, with recruitment assistance from facility administrators and eligibility screening by the research team. Semistructured face-to-face interviews were conducted between September and November 2025. Data were analyzed using framework analysis.

**Results:**

Framework analysis produced 4 analytical domains: real-world care needs, limited facilitating conditions, major anticipated barriers, and preparatory pathways for implementation. Participants expected robots to support safety monitoring and risk warning, standardized checking and management, repetitive physical tasks, and basic interaction. Although some openness to technology and organizational support existed, these facilitating conditions were insufficient for routine implementation. Key barriers included unclear human-robot collaboration models, inadequate organizational capacity, potential new care risks, and weak external support. Participants emphasized organizational assessment, contextual adaptation, pilot testing, and structured training as essential preparatory steps. Emotional support functions were also valued for alleviating caregivers’ emotional burden in high-intensity care work.

**Conclusions:**

Openness to technology, supportive organizational climates, and positive policy and social signals provided an initial foundation for considering robot integration. However, uncertainties remained around human-robot collaboration, risk management, organizational capacity, and concrete external support. Organizational assessment, contextual adaptation, real-world pilot testing, and practical training warrant further evaluation before wider implementation.

## Introduction

Population aging is creating a sustained increase in the demand for long-term care services [1]. In China, smaller family structures and changes in family caregiving resources [2], together with the growing care needs of older adults with advanced age, functional impairment, and cognitive impairment [3], have increased the importance of long-term care facilities. However, the growing demand for institutional care coincides with a relative shortage of frontline care workers, while high workloads and turnover risks further challenge the stability of the institutional care workforce [4]. Against this background, China has increasingly promoted smart eldercare through national action plans and pilot demonstration programs [5], while smart care robots have attracted attention as a potential means of supporting frontline care, alleviating care-related pressures, and improving care quality [6,7].

Smart care robots are service robots designed for older adult care settings, with varying degrees of autonomy and human-robot interaction capabilities, and are intended to support daily living, care activities, and health-related needs [8,9]. According to their primary functions, smart care robots can generally be classified as assistive robots or socially assistive robots [7]. Assistive robots, such as Lio (F&P Robotics AG) and Care-O-bot (Fraunhofer Institute for Manufacturing Engineering and Automation IPA), primarily support physical or task-oriented care activities, including transfers, mobility assistance, medication support, and the delivery of items [8]. Socially assistive robots, such as PARO (National Institute of Advanced Industrial Science and Technology) and Pepper (SoftBank and Aldebaran Robotics), mainly provide emotional, social, and cognitive support through human-robot interaction [10]. Existing evidence suggests that smart care robots may improve older adults’ emotional well-being, interpersonal communication, and quality of life, although their effects vary according to robot type, application setting, and mode of intervention [11].

Nevertheless, smart care robots remain largely at the stage of exploratory application or small-scale piloting [12,13], and their implementation in real-world long-term care facilities continues to face multiple challenges [14,15]. Recent research has increasingly emphasized a multistakeholder perspective [16,17], recognizing that technology adoption in long-term care involves not only older adults but also care workers and organizational stakeholders. As direct care providers, nursing assistants are familiar with residents’ practical care needs [18,19], as well as institutional care workflows and resource conditions [14,20]. They are therefore well positioned to identify the conditions required to integrate smart care robots into long-term care practice [21,22].

Existing studies have mainly examined nursing assistants’ attitudes towards existing robots or predefined functions, their functional expectations [21,23], and ethical concerns [14], or have used the Technology Acceptance Model (TAM) and the Unified Theory of Acceptance and Use of Technology (UTAUT) to investigate individual-level acceptance factors such as perceived usefulness and perceived ease of use [24-26]. Some studies have begun to emphasize identifying technological requirements from actual care work and frontline workers’ needs [8,27]. Technology acceptance research further suggests that the factors influencing adoption should be examined in relation to specific technologies and application settings [28,29]. Implementation science research indicates that the integration of technology into care practice is also shaped by multilevel contextual factors, including workflows, organizational readiness, resource conditions, and external support [30,31]. However, systematic investigation of nursing assistants’ expectations of robot functions, conditions for integration into practice, and preimplementation preparations remains limited [21,32]. Identifying these factors before formal implementation may improve the fit between technology and care practice while reducing subsequent trial and error costs and resource waste [33,34].

Accordingly, this qualitative descriptive study aimed to explore nursing assistants’ expectations regarding the functions of smart care robots, the conditions influencing their integration into routine care, and the preparations required before implementation in long-term care facilities. The study addressed 3 research questions:

1. What care functions do nursing assistants expect smart care robots to perform?
2. What barriers and facilitating conditions do they perceive as influencing the integration of these robots into routine care?
3. What preparations do they consider necessary before implementation?

## Methods

### Design

This study used a qualitative descriptive design. Qualitative description is a naturalistic and pragmatic approach that seeks to provide clear, comprehensive, and data-near accounts of participants’ experiences and perspectives in everyday terms [35,36]. This design was appropriate for describing nursing assistants’ preimplementation expectations of smart care robots without seeking to generate a formal theory or test hypotheses. The interview data were analyzed using framework analysis, as described below. The study was reported in accordance with the Consolidated Criteria for Reporting Qualitative Research (COREQ; Checklist 1) [37].

### Conceptual Positioning and Analytical Orientation

The TAM and the UTAUT primarily directed attention to individual-level factors associated with technology acceptance [28,38], whereas the Consolidated Framework for Implementation Research (CFIR) further sensitized the research team to organizational, external, and implementation-related contextual influences [39,40]. These frameworks were used as sensitizing concepts rather than coding templates, and the data were not formally mapped onto their predefined constructs. Consistent with framework analysis, the analysis combined deductive and inductive elements: the research questions and interview guide informed 3 initial overarching analytical domains, whereas the specific codes, analytical categories, and their boundaries were developed and iteratively refined through engagement with participants’ accounts.

### Participants and Settings

The target participants were nursing assistants working in long-term care facilities. In this study, nursing assistants were defined as paid workers who provided direct care in long-term care facilities but were not licensed nurses. Their responsibilities could include assistance with activities of daily living, basic care, rehabilitation support, psychosocial support, and care-related observation. The inclusion criteria were: (1) direct involvement in frontline care, (2) provision of informed consent and voluntary agreement to participate, and (3) adequate ability to communicate and express their views. The exclusion criteria were: (1) managerial, administrative, or support staff not directly involved in care; and (2) interns, trainees, or short-term part-time workers.

Between September and November 2025, purposive sampling was used to recruit participants from long-term care facilities in Chengdu, Sichuan Province, China. Participating facilities were identified from the official list of registered long-term care facilities published by the Chengdu Civil Affairs Bureau. To include facilities with different organizational backgrounds, the research team purposively selected 18 facilities from this list based on facility size, operating model (public, private, or publicly built and privately operated), and geographical location (urban or suburban), and sent each facility an invitation describing the study purpose, participant eligibility criteria, and data collection procedures. Five facilities responded and agreed to participate, and all were included in subsequent participant recruitment. The participating facilities had 65‐720 beds and employed 8‐102 nursing assistants. Further facility characteristics are presented in Table S1 in Multimedia Appendix 1.

The 5 participating facilities employed a total of 201 nursing assistants. With assistance from facility administrators, 41 potentially eligible nursing assistants were identified for formal screening, with consideration given to the eligibility criteria and variation in sex, age, educational background, professional title, and years of care experience. The research team screened all 41 individuals and confirmed that they met the eligibility criteria. Of these, 34 agreed to participate, and 7 declined. Interviews with consenting participants were scheduled sequentially according to their work schedules and availability. Three of the 34 consenting participants subsequently did not complete an interview because of scheduling conflicts or personal reasons. The remaining 31 nursing assistants completed interviews and were included in the analysis.

The final sample of 31 participants was pragmatically determined by operational constraints during the planned data collection period, including the number of eligible nursing assistants identified through the participating facilities, their willingness and availability to participate, and scheduling constraints related to their care work. Participant characteristics, including sex, age, educational background, professional title, years of care experience, daily working hours, monthly days off, and night-shift frequency, are presented in Table 1. Participants’ previous exposure to care robots was also recorded to clarify whether their accounts were based on direct use, observation in real-world settings, or indirect exposure.

**Table 1.** Participant characteristics.

ID	Sex	Age (years)	Education	Professional title	Work experience (years)	Daily working hours (hours)	Monthly days off (days)	Night shift frequency (times/month)
P1	Female	55	Primary school	None	2	8	4	3‐4
P2	Female	49	Junior high school	Junior	5	8	4	3‐4
P3	Male	49	Junior high school	Junior	5	8	4	3‐4
P4	Female	54	Junior high school	Intermediate	5	8	4	None
P5	Female	55	Primary school	None	7	14	4	3‐4
P6	Female	54	Junior high school	Junior	6	14	4	3‐4
P7	Female	53	Primary school	Junior	5	14	4	3‐4
P8	Female	49	Junior high school	Junior	2	12	4	3‐4
P9	Male	56	Junior high school	Junior	6	10	4	3‐4
P10	Female	54	Junior high school	Junior	2	10	4	None
P11	Female	30	Junior high school	Junior	1	12	4	3‐4
P12	Female	55	Junior high school	Intermediate	7	10	4	3‐4
P13	Male	56	Junior high school	Junior	1	10	4	3‐4
P14	Female	48	Junior high school	Junior	4	10	4	3‐4
P15	Male	55	Junior high school	Junior	5	10	4	3‐4
P16	Female	49	Junior high school	Junior	5	9	8	6‐7
P17	Female	38	Primary school	Intermediate	5	9	8	6‐7
P18	Female	51	Junior high school	Junior	5	9	8	6‐7
P19	Female	57	Junior high school	Intermediate	11	9	8	6‐7
P20	Female	52	Junior high school	Intermediate	7	9	8	6‐7
P21	Female	51	Senior high school	Senior	9	9	8	6‐7
P22	Female	48	Primary school	Junior	1	14	4	None
P23	Female	57	Junior high school	Junior	10	14	4	None
P24	Female	53	Junior high school	Senior	6	14	4	None
P25	Female	55	Primary school	None	3	14	4	None
P26	Male	48	Senior high school	Intermediate	6	12	4	7‐8
P27	Male	22	Junior college	Senior	1	12	4	None
P28	Male	25	Junior college	Senior	2	12	4	7‐8
P29	Male	45	Senior high school	Intermediate	6	12	4	7‐8
P30	Female	22	Junior college	Senior	2	12	4	7‐8
P31	Female	47	Junior high school	Junior	1	12	4	7‐8

### Data Collection

Data were collected through semistructured, face-to-face interviews conducted between September and November 2025. All formal interviews were conducted by the first author (YA), a female graduate student who had received training in qualitative research methods. The interviewer had no prior relationship with the participants. The interview guide was developed from the study aim and research questions, informed by previous literature on care robots, health technology adoption, and technology implementation in long-term care facilities, and finalized through research team discussion.

Before formal data collection, 3 nursing assistants were recruited separately to participate in pilot interviews assessing the clarity, relevance, and comprehensibility of the interview questions. Based on their feedback, the wording and order of several questions were revised, resulting in the final interview guide presented below:

1. Could you describe your main duties in the long-term care facility?
2. In your daily care work, which tasks do you find most burdensome or requiring the most attention?
3. How do you view the application of smart care robots in long-term care facilities?
4. Based on your work experience, in which care tasks do you think smart care robots would be suitable or unsuitable, and why?
5. What factors do you think may influence the integration of smart care robots into long-term care practice?
6. If long-term care facilities were to consider introducing smart care robots in the future, what conditions do you think would be needed in terms of organizational support, training, and external support?
7. What other views or suggestions do you have regarding the future application of smart care robots in long-term care facilities?

The 3 pilot participants were recruited separately from the same 5 facilities before formal participant screening began and were not drawn from the 41 individuals identified as eligible for formal recruitment. They were therefore not counted among the 41 eligible individuals and were excluded from Table 1 and all other formal study sample counts. The pilot interview data were used only to refine the interview guide and were not included in the formal analysis.

Interviews were scheduled at times convenient for participants and conducted in relatively quiet, private spaces within the facilities, such as activity rooms or nursing assistants’ break rooms, to minimize interruptions and allow participants to express their views privately. Before each interview, the researcher (YA) introduced her role and research background and explained the study purpose, confidentiality arrangements, and voluntary nature of participation. Written informed consent was obtained before the interview, and interviews were audio-recorded with participants’ permission. All interviews were conducted face-to-face and one-to-one and lasted approximately 20‐40 minutes. The researcher recorded relevant nonverbal cues, including facial expressions, body posture, and changes in tone. Probing, clarification, and reflective summarizing were used to explore the meanings and contexts of participants’ accounts. The interviewer sought to minimize leading questions and used follow-up prompts primarily to clarify or confirm participants’ intended meanings.

### Data Analysis

Within 24 hours of each interview, the first author (YA) listened repeatedly to the audio recording and transcribed it verbatim. The second author (FZ) checked each transcript against the recording for accuracy. Unclear content was discussed by the research team and, where necessary, clarified with the relevant participant. Verified transcripts were deidentified and imported into NVivo 15 (Lumivero), and the recordings were securely deleted after their accuracy had been confirmed. The data were analyzed using framework analysis, which was appropriate for addressing focused, applied research questions and enabled systematic comparison within and across cases and analytical categories while retaining openness to unanticipated, context-specific findings [41]. Following established guidance, the analysis involved 6 iterative stages after transcription: familiarization, initial coding, development of a working analytical framework, application of the framework, charting, and interpretation [42].

YA and FZ independently read and line-by-line coded all 31 transcripts. The research questions and interview guide provided 3 initial overarching analytical domains: (1) expected care functions of smart care robots, (2) conditions influencing their integration into routine care, and (3) preparations required before implementation. Facilitating and hindering factors, organizational support, training, and external support were acknowledged as a priori areas of interest, while data-driven codes were generated within and beyond these domains to capture unanticipated care needs, implementation concerns, and contextual factors. Illustrative coding examples are provided in Table S2 in Multimedia Appendix 1. Based on the initial coding, conceptually related codes were grouped into working analytical categories. Although the overarching domains were informed by the research questions and interview guide, the specific codes, category labels, definitions, and boundaries were developed and refined through engagement with the data. In particular, accounts of existing enabling conditions were differentiated from substantive implementation barriers, dividing the initial domain of influencing conditions into “limited facilitating conditions” and “major anticipated barriers.” Examples of this analytical progression are presented in Table S3 in Multimedia Appendix 1.

The working framework was iteratively revised by adding, merging, dividing, renaming, or redefining categories when necessary. Previously coded transcripts were revisited after substantive revisions to ensure consistent application. The revised framework was then applied to all transcripts for indexing, and a matrix was constructed with participants in rows and analytical categories in columns. Each cell contained a concise summary of the participant’s relevant account, with the participant identifier retained to support comparison with the original transcript. An excerpt from the matrix is provided in Table S4 in Multimedia Appendix 1. Interpretation involved comparing similarities, differences, contrasting accounts, and relationships across cases and categories. The final framework comprised 4 analytical domains and 15 analytical categories, as presented in Table S5 in Multimedia Appendix 1. YA and FZ jointly developed and revised the working analytical framework and interpreted the matrix. They resolved differences in coding or category boundaries through discussion. When additional adjudication was required, the third researcher (XX) reviewed the relevant transcript excerpts, codes under discussion, category definitions, and related analytical records. Final decisions were reached through team discussion and documented in the analytic audit log. Successive framework versions, matrices, analytic notes, and discussion records were retained as an audit trail.

### Ethical Considerations

This study was conducted in accordance with the Declaration of Helsinki and was approved by the Biomedical Ethics Review Committee of West China Hospital, Sichuan University (No. 20252018). Written informed consent was obtained from all participants. To protect privacy, participants’ names and other identifiable information were removed during the research process and replaced with study codes.

### Rigor

Trustworthiness was supported in accordance with the criteria of credibility, transferability, dependability, and confirmability [43]. All 31 transcripts were independently coded by both YA and FZ. Coding disagreements were resolved through discussion and, when necessary, referred to XX for additional adjudication. To enhance credibility, member checking was conducted in February 2026 by the first author (YA), who had conducted the interviews, after initial coding and development of the working analytical framework but before its finalization. Using the contact details provided during recruitment, the first author attempted to contact all 31 participants by telephone to explain the purpose and procedure. Participants reached by telephone were subsequently sent via WeChat (Tencent) a summary of the preliminary findings relevant to their accounts, including the key points from their interviews and the research team’s preliminary interpretation. They were informed that they could assess whether the summary accurately reflected their intended meanings and main views and provide any corrections, clarifications, or additions within 7 days. For participants who could not be reached by telephone or did not respond via WeChat within the specified timeframe, the relevant summary was presented and discussed verbally during a face-to-face review. All 31 participants completed member checking within 10 days, with 14 completing it via WeChat and 17 through face-to-face follow-up. Participants generally considered the summaries consistent with their views, and no participant requested substantive corrections, although some reiterated points they considered important. Based on this feedback, no substantive changes were made to the working analytical framework. Following each completed review, the first author (YA) promptly documented the review process and feedback in an anonymized member-checking summary log, as detailed in Table S6 in Multimedia Appendix 1. Transferability was supported through detailed reporting of the study setting, participant characteristics, and research procedures. To support dependability and confirmability, the research team maintained an audit trail documenting data management, coding decisions, revisions to the analytical framework, and analytic discussions. All transcripts were prepared and analyzed in Chinese, the participants’ primary language. The findings and selected quotations were translated into English only after the analysis had been completed to preserve the meanings and contexts of participants’ original accounts as far as possible.

## Results

### Overview

Most participants had no direct hands-on experience with care robots. Their accounts were based primarily on information from news or social media and observations of robots in care-related or public service settings. Only a small number reported direct experience with robots. Participants’ types of exposure and the robots involved are presented in Table 2. Framework analysis of the interviews with 31 nursing assistants produced 4 analytical domains and 15 analytical categories (Figure 1; Table S5 in Multimedia Appendix 1). The first domain described participants’ expectations regarding the care functions of smart care robots. The second and third domains captured the limited facilitating conditions and major anticipated barriers associated with integrating robots into routine care practice. The fourth domain outlined the preparations participants considered necessary before formal implementation. To enhance transparency, Table 3 summarizes the number of participants whose accounts contributed to each analytical domain and category [44,45]. These numbers are provided only to describe the breadth of participant accounts informing each analytic category within this sample and should not be interpreted as statistical prevalence, inferential results, or indicators of the relative importance of analytical domains or categories [46,47].

**Table 2.** Participants’ prior exposure to care robots and related robot types.

ID	Exposure to care robots	Robot type
P1	Indirect exposure through news or social media	—^a^
P2	Indirect exposure through news or social media	—
P3	Direct experience	Assistive robots (guiding and meal delivery)
P4	Real-world observation	Assistive robots (rehabilitation)
P5	Indirect exposure through news or social media	—
P6	Indirect exposure through news or social media	—
P7	Indirect exposure through news or social media	—
P8	Direct experience	Social robots
P9	Indirect exposure through news or social media	—
P10	Indirect exposure through news or social media	—
P11	Real-world observation	Assistive robots (meal delivery and cleaning)
P12	Indirect exposure through news or social media	Social robots and assistive robots (feeding assistance)
P13	Real-world observation	Assistive robots (meal delivery)
P14	Real-world observation	Assistive robots (cooking and household tasks)
P15	Direct experience	Social robots
P16	Real-world observation	Assistive robots (patient transfer)
P17	Real-world observation	Assistive robots (medication management)
P18	Indirect exposure through news or social media	—
P19	Real-world observation	Assistive robots (meal delivery)
P20	Indirect exposure through news or social media	—
P21	Direct experience	Assistive robots (cleaning support)
P22	Indirect exposure through news or social media	—
P23	Indirect exposure through news or social media	—
P24	Indirect exposure through news or social media	—
P25	Indirect exposure through news or social media	—
P26	Real-world observation	Social robots
P27	Real-world observation	Social robots and assistive robots (transfer)
P28	Real-world observation	Social robots
P29	Real-world observation	Social robots
P30	Real-world observation	Social robots
P31	Indirect exposure through news or social media	—

a Not applicable.

**Figure 1.**
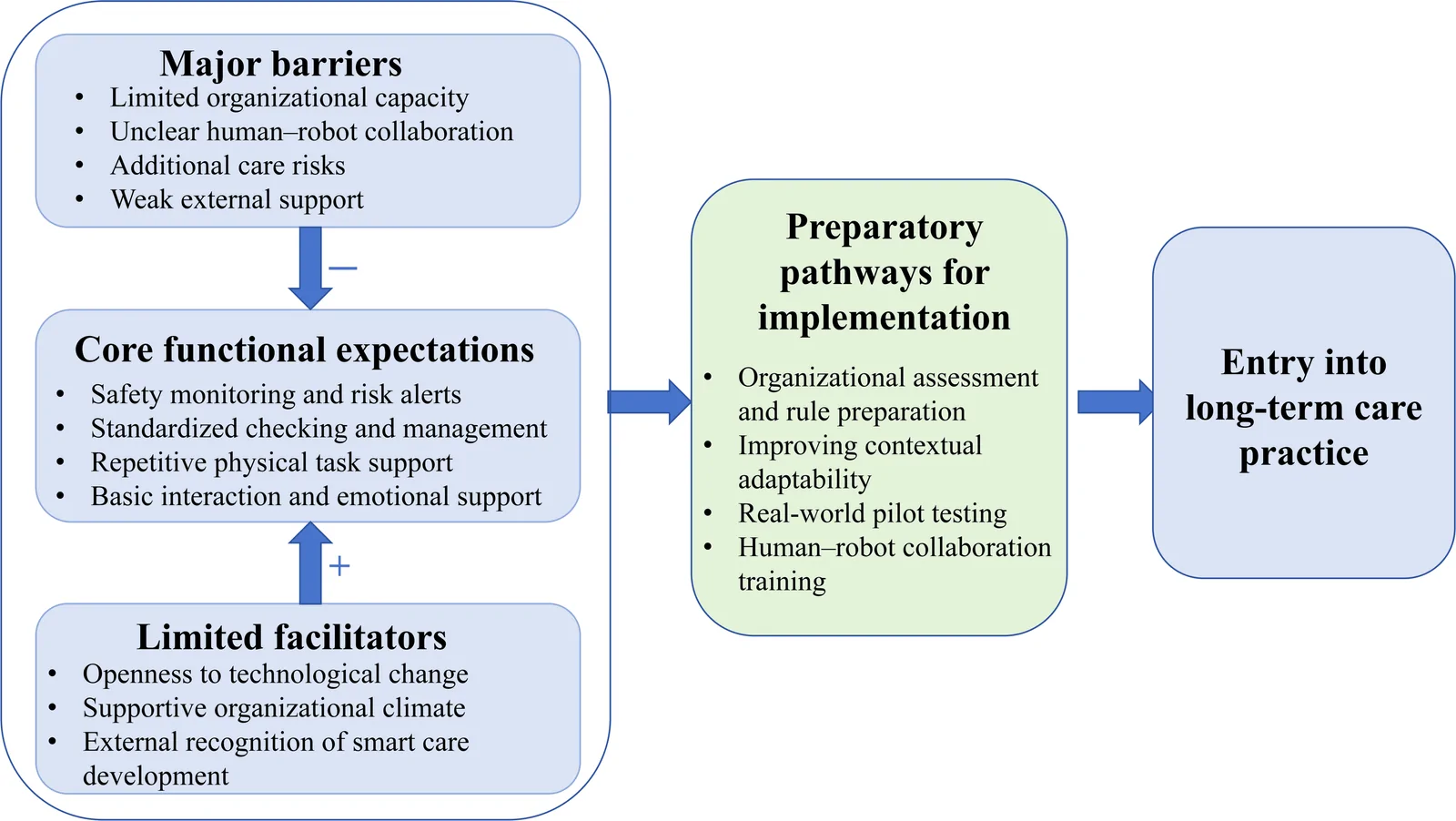
Relationships among the 4 analytical domains regarding the integration of smart care robots into care practice in long-term care facilities.

**Table 3.** Breadth of participant contributions across analytical domains and categories (N=31).

Analytical domains and categories	Participants contributing, n	Participant IDs
Real-world care needs		
Support for safety monitoring and risk warning	11	P3, P4, P7, P8, P11, P15, P20, P24, P27, P28, P30
Support for standardized checking and management	8	P14, P16, P17, P19, P21, P26, P28, P29
Support for repetitive physical tasks	12	P1, P2, P3, P4, P5, P8, P10, P11, P12, P23, P24, P27
Basic interaction and emotional support	9	P2, P3, P7, P11, P13, P16, P28, P29, P30
Limited facilitating conditions		
Nursing assistants’ openness to technological change	9	P3, P9, P17, P19, P26, P27, P28, P30, P31
A supportive organizational climate and inclusive management practices	12	P3, P7, P9, P10, P12, P14, P15, P16, P17, P25, P27, P30
External recognition of the value and potential of intelligent care	8	P17, P21, P23, P24, P26, P27, P29, P31
Major anticipated barriers		
Inadequate organizational resources and implementation conditions	7	P3, P6, P8, P14, P18, P21, P24
Unclear models of human-robot collaborative care	9	P3, P6, P11, P13, P14, P22, P23, P25, P29
Potential new care risks introduced by technology use	12	P2, P3, P5, P9, P10, P15, P16, P22, P25, P26, P29, P31
Weak external support conditions	14	P3, P4, P5, P6, P7, P9, P12, P19, P20, P21, P22, P28, P29, P31
Preparatory pathways for implementation		
Organizational assessment and rule preparation	8	P8, P10, P18, P23, P26, P27, P28, P31
Improving contextual adaptability	11	P11, P13, P15, P16, P18, P19, P21, P27, P28, P29, P30
Pilot application in real-world care settings	10	P2, P3, P8, P10, P12, P13, P15, P22, P23, P24
Training for human-robot collaborative care	9	P4, P7, P11, P17, P18, P20, P21, P27, P29

### Analytical Domain 1. Real-World Care Needs: Nursing Assistants’ Core Functional Expectations of Smart Care Robots

#### Support for Safety Monitoring and Risk Warning

Participants expected smart care robots to intervene at critical points before adverse events occurred, through functions such as dynamic following and real-time reminders, so as to reduce negative outcomes. They also hoped that such support could protect older adults’ safety while reducing excessive restriction and preserving greater freedom of movement.

As soon as you turn away, some older adults try to stand up. It would be good if a smart care robot could stay with them and follow them around, and even chat with them a little. Otherwise, if we keep restraining them all the time, we would feel bad about it.

[P24]

They also hoped that robots could follow instructions to enhance focused observation:

It would be good if the robot could follow instructions and move to each bedside, especially to observe and check on agitated older adults.

[P4]

In addition to behavioral monitoring, participants also expected smart care robots to detect abnormalities in physiological indicators.

Some older adults have hypertension. When they stand up, their blood pressure may change, which could cause them to fall.

[P28]

Robots were also expected to detect and respond to environmental risks.

Many older adults think they can still walk, but their legs are stiff and their eyesight is poor. If they miss a step, they may fall. It would be good if a robot could remind them or call me for help.

[P3]

In addition to daytime care, participants also expected smart care robots to help ensure older adults’ safety at night while minimizing disturbance and preserving sleep continuity.

At night, we have to check the rooms every two hours. Once older adults are woken up, it is hard for them to fall asleep again. If the robot had something like far-infrared sensing, it could help us check their physical condition without waking them, and it would be more accurate.

[P15]

Participants also hoped that robots could respond quickly to ineffective calls at night.

Some older adults do not sleep at night and keep pressing the call bell. If we are not quick enough, they start shouting and making a fuss, which disturbs other residents’ sleep. Every time, we still have to go over and check on them.

[P20]

In addition, bed rail status was viewed as an important risk point in night-time care.

Some older adults who are still mobile secretly lower the bed rails, or forget to put them back after going to the toilet. That can easily lead to a fall from the bed, so we have to watch for it all the time, and it is hard to guard against.

[P20]

It is the same with the bed rails, they often lower them secretly. It is manageable during the day because there are more of us, but at night we really cannot keep up.

[P28]

#### Support for Standardized Checking and Management

Participants expected smart care robots to support more accurate checking and management, thereby reducing missed checks and errors. This included regularly checking the expiry dates of food items.

In our facility, we check the expiry dates of the snacks older adults keep in their wardrobes or storage areas every week, but sometimes things are still missed.

[P21]

They also hoped that robots could assist with medication management and reduce the pressure associated with errors.

Everyone’s medication is different, and it can change with their condition. When medicines are distributed manually, we are always worried about mistakes. We hope a robot could help sort them out by the right time, the right dose, and for the right person.

[P17]

#### Support for Repetitive Physical Tasks

Participants also expected smart care robots to take on repetitive physical tasks, thereby reducing nursing assistants’ physical burden and improving older adults’ comfort and the timeliness of care.

Work that requires skill is manageable, but sometimes it is the physical demands that we really cannot keep up with.

[P5]

This included repetitive and standardized exercise support.

For rehabilitation exercises such as leg lifting, leg stretching, and ankle or wrist movements, smart care robots could completely take over. That would save manpower, and robots could do it more consistently and to a better standard.

[P27]

Participants also hoped that robots could assist with lifting and transfers.

Semi-dependent older adults need to be transferred many times a day between the bed and the wheelchair. Doing this so often makes our backs hurt.

[P24]

Once, when I was moving a heavier older adult, both of us fell. A robot could probably lift any weight, and the force it used would be more even.

[P1]

In addition, participants hoped that robots could improve the timeliness of care.

Some older adults often want to sit up, but sometimes we are too busy and they feel embarrassed to call us. If it were a robot, then whenever they called, it could go and help lift them up straight away.

[P2]

#### Basic Interaction and Emotional Support

Several participants believed that smart care robots could engage older adults in simple interactions and respond to their interests, but could not replace genuine emotional communication.

Tasks like telling stories, playing music, having simple conversations, or answering questions about the weather are completely manageable for a robot.

[P28]

Smart care robots were also expected to increase older adults’ participation.

Every day, we arrange for older adults to follow exercise videos, but some just sit there and do not want to move. A smart care robot could go over to them like we do, encourage them, and guide them through the exercises. That would be better than just using a television.

[P30]

Participants also valued the broader knowledge base that robots might offer to match older adults’ interests.

Sometimes, when we chat with older adults, we cannot know everything. A smart care robot could answer all kinds of questions they ask.

[P3]

At the same time, nursing assistants drew a clear boundary in role allocation.

Only communication between people carries real emotion. When older adults are in a low mood, they would not want to confide in a robot. The smart care robot can help me with other tasks, and I will talk with the older adult.

[P16]

Notably, nursing assistants’ expectations of the emotional support functions of smart care robots were more closely related to easing their own emotional burden. When caring for older adults with cognitive impairment, participants expected robots to provide stable and repetitive basic responses.

He keeps asking the same questions. No matter what you say, you still have to keep responding, and that can make me feel a bit irritated. A robot has no emotions and can keep giving the same response over and over.

[P2]

Participants also expected robots to reduce missed care and thereby indirectly relieve psychological burden.

When we are too busy, some older adults may not be cared for as carefully as they should be. I feel guilty about that, but there is not much I can do. If there were a smart care robot, it would definitely help.

[P11]

### Analytical Domain 2. Limited Facilitating Conditions: Factors Providing a Basis for the Entry of Smart Care Robots Into Practice

#### Nursing Assistants’ Openness to Technological Change

Nursing assistants viewed smart care robots as new tools with practical value that could be learned and mastered. This openness to technological change provided an initial basis for their entry into long-term care facilities. Participants also expressed positive expectations regarding their future role in robot-assisted care, for example.

Our nursing staff here are mostly older; I would definitely be the main operator. If the institution introduces robots, I would be the first to learn how to use them and then teach the older colleagues.

[P30]

From their understanding of the complexity of care work and its associated risks, participants recognized the supportive value of smart care robots.

Hygiene involves privacy protection; walking carries the risk of falls; feeding means preventing choking; and older adults can easily become ill when the weather changes. Care work is not as easy as outsiders think, and there are many areas where robots could help us strengthen care.

[P9]

Participants also believed that their existing care knowledge would help them acquire new technological skills.

We have a background in care, so it should be easier for us to learn and understand it.

[P26]

Participants further highlighted the potential of human–robot collaboration to enhance care quality.

Now many robots are equipped with radar and similar functions, which can provide real-time feedback on residents’ conditions so that I can respond in time.

[P27]

Robots have machine learning and adaptive capabilities, and I can input residents’ care preferences so that they can learn them.

[P28]

In addition, participants emphasized mutual learning between humans and robots.

Smart care robots have their own way of operating, and we have human care experience. We can learn from each other and do the job better.

[P19]

#### A Supportive Organizational Climate and Inclusive Management Practices

A supportive organizational climate and inclusive management, although not specifically established for new technology use, provided some organizational buffer for the uncertainty associated with integrating smart care robots into practice. Accessible and fair management was reflected in participants’ accounts.

When we cannot solve a problem, we go to the manager. He is very willing to help and considers everyone’s interests fairly, without favoring anyone.

[P16]

Management was also described as oriented towards correction and improvement rather than punishment.

When mistakes happen, the facility does not punish us just for the sake of punishment. They talk with us and provide training to help us avoid making the same mistake again.

[P25]

A culture of teamwork and experience sharing further provided a basis for discussing problems and adapting practice after the introduction of new technology.

If I cannot communicate well with an older adult I am responsible for, my colleagues will help and try talking with that person instead.

[P9]

Our facility has a monthly sharing meeting where everyone talks about the care problems they have encountered. If smart care robots are introduced in the future, we could definitely discuss them there as well.

[P27]

Recognition of labor value and emotional support was also evident.

The director really understands our work and is considerate of us. She usually takes the initiative to check in on us, and once during a holiday she gave us 200 yuan to give to our parents. That made up, to some extent, for the guilt we felt inside, and it also made us feel that this work was truly worthwhile.

[P14]

#### External Recognition of the Value and Potential of Intelligent Care

Positive signals from the broader environment regarding the use of intelligent technologies in eldercare provided some directional support for the introduction of smart care robots into long-term care facilities. This was reflected in participants’ recognition of the broader trend towards intelligent care.

Society is developing, technology is advancing, and there is constant discussion about smart eldercare as the way forward. It feels like eldercare will definitely move in this direction.

[P26]

Participants also referred to positive expectations about care quality.

In external publicity, they always say that smart care robots do not get tired, do not have emotions, and can keep service quality at a stable standard.

[P21]

In addition, some understood the value of robots as reducing workload rather than replacing staff.

They say that if robots can share some of the work, we will be able to spend more time with older adults, not that they are meant to replace us.

[P17]

### Analytical Domain 3. Major Anticipated Barriers: Key Conditions Hindering the Entry of Smart Care Robots Into Practice

#### Inadequate Organizational Resources and Implementation Conditions

Whether smart care robots can be integrated into practice in long-term care facilities depends not only on the technology itself, but also on whether facilities have the basic conditions needed to absorb new technologies. Participants described a lack of time and space to learn and adapt to new technologies.

This is not like a hospital, where doctors and nurses have clearly divided responsibilities. Here, we have to take care of almost everything related to the older adults. What we do each day, and what we do first, often depends on what they need at that moment. By the end of the day, there is almost no real free time.

[P14]

Responsibility was also described as highly individualized, leaving little room for risk sharing.

Here, each of us is assigned specific older adults and remains responsible for them until they die. Care is not organized on a rotating basis. Other staff are not very familiar with their situations, so handovers are difficult. Apart from emergencies, people rarely take leave.

[P24]

Participants further noted insufficient support and weak standardization during transitional periods.

The adjustment period with newly admitted older adults is when conflicts are most likely to happen. Management worry that family complaints will affect occupancy, so the problem is mainly blamed on the nursing assistant. When I first started this job, an experienced nursing assistant was training me. Once, both of us were fined before I even knew that what we had done was against the rules.

[P8]

#### Unclear Models of Human-Robot Collaborative Care

Once smart care robots are introduced into long-term care facilities, care tasks previously performed solely by nursing assistants are expected to shift towards human-robot collaboration. However, this transition currently lacks a clear supporting model. Participants reported uncertainty about how smart care robots would be incorporated into routine care processes.

A smart care robot would be like a new colleague, but would the workflow be different? Would I also need to spend time helping it get familiar with the older adults before formal care begins?

[P23]

The scope of human responsibility was also unclear.

If you just press a button and the robot goes to do the task, then what would I be doing? When I am caring for older adults, would I also have to supervise whether it is going to malfunction?

[P6]

In addition, there were no clear standards for how changes in workload and return would be assessed.

Right now, I care for seven or eight older adults. If robots are introduced, they will definitely ask me to care for more people. So has my workload actually changed, and how would my pay change?

[P29]

#### Potential New Care Risks Introduced by Technology Use

An important barrier to the integration of smart care robots into long-term care facilities was the concern that, if they lacked the ability to identify actual needs and respond flexibly within specific care situations, they might introduce new risks into practice. Participants noted that robots might not be able to interact effectively with some older adults.

Many older adults have poor eyesight and hearing, so they may not be able to trigger a response from a smart care robot. Not every older adult can use a robot.

[P3]

Participants also questioned whether robots could recognize needs with an emotional component.

Some older adults cry out in pain even when you touch them very lightly. Sometimes they really are uncomfortable, and sometimes they are just in a bad mood and want attention. How would a smart care robot tell the difference?

[P22]

They further raised concerns about robots’ ability to interpret verbal instructions together with physical status.

He wants to stand up, but says ‘sit down’ instead. Could the robot understand that?

[P29]

If an older adult gives the wrong instruction, a mistake in a small task such as fetching something might not matter too much. But if it involved something more serious, the consequences would be unimaginable.

[P31]

Participants also doubted whether robots could adjust in time according to the specific situation.

If a robot is helping an older adult put on trousers, it cannot judge whether the person’s leg can bend or lift, or whether this might cause a fracture or injury. It would just follow the instruction rigidly.

[P29].

In addition, participants worried about the possibility of direct harm if the robot lost control.

What if the robot does not follow instructions and ends up hurting an older adult? Older adults are not as quick to react as we are, and they may not even be able to get out of the way.

[P16].

#### Weak External Support Conditions

Weak external support conditions undermined institutional trust in smart care robots and increased concerns before their integration into care practice. Participants described uncertainty about the authenticity and detail of external information.

I saw a video online showing a robot hitting an older adult. Is that real or fake?

[P7]

They keep saying that intelligent technology can reduce our care burden, but we still do not know exactly how smart care robots would do that.

[P12]

They also noted the lack of product standards and quality assurance tailored to eldercare settings.

Machines will definitely malfunction, just like people make mistakes. But for smart care robots used to care for older adults, there should be clear quality standards. You cannot just let any robot enter a facility.

[P21]

Responsibility attribution was also seen as unclear.

If it detects that an older adult is in danger but does not issue an alarm, is that the nursing assistant’s fault for not checking in time, or the smart care robot’s fault? The robot itself cannot take responsibility.

[P31]

In addition, from the perspective of older adults’ family members, the consequences of technology use would still ultimately be borne by the facility.

If something goes wrong, who will the family blame? They will definitely come to the facility and say, ‘We entrusted the older adult to you, and then you handed them over to a robot.

[P6]

### Analytical Domain 4. Preparatory Pathways for Implementation: Preimplementation Work to Support the Effective Integration of Smart Care Robots Into Practice

#### Organizational Assessment and Rule Preparation

Before smart care robots are introduced, implementation conditions need to be clarified and assessed in order to reduce uncertainty in subsequent use. Participants emphasized the need for clear rules and procedures.

There should be clear operating procedures in place first. If we are left to act based on our own judgment without knowing what is right or wrong, that would be a major concern.

[P8]

Management should conduct comprehensive assessments and make decisions in light of the facility’s actual conditions.

Management needs to investigate first and think it through carefully, to decide whether the robot can be integrated into our team, whether it can work well with everyone, and whether it can help care for older adults properly.

[P10]

Participants further suggested a gradual, lower-risk introduction.

It would be better to start with simple supportive tasks that do not involve direct contact with older adults. Not only do we need time to adapt, but older adults also need time. It should be introduced gradually.

[P27]

#### Improving Contextual Adaptability

To improve the feasibility and practical value of smart care robots in real-world care, their ability to adapt to complex care situations also needs to be strengthened, rather than remaining limited to passively carrying out instructions. Participants expected robots to be able to attend to multiple tasks and conditions at the same time.

When we turn an older adult, we also need to check whether the diaper needs changing, whether there are any pressure sores, and what the person’s physical condition is like at that moment. Smart care robots should be able to do the same.

[P18]

They also expected robots to be able to recognize unusual situations and follow up on possible causes.

When an older adult behaves differently from usual, we need to find out why. For example, if an older adult does not want to eat, a smart care robot should ask further questions about whether they feel physically or emotionally uncomfortable, rather than just leaving.

[P16]

In addition, participants highlighted the need for personalized adaptability.

With older adults with cognitive impairment, you need to understand their habits and the things that require attention. If communication is difficult, then you should at least respond to their emotions.

[P13]

#### Pilot Application in Real-World Care Settings

Pilot use in real-world care settings was considered important for assessing the usability, safety, and contextual fit of smart care robots. They wanted to observe the full care process firsthand.

If I could see with my own eyes the whole process of it caring for an older adult, and see that it could do it well and skilfully, then I would definitely feel reassured.

[P10]

Key tasks were also seen as needing focused validation.

You can tell whether a robot is really useful just from one or two details, for example, by asking it to assist with turning an older adult.

[P23]

In addition, participants emphasized the need for adjustment during use.

There needs to be a running-in period to identify its problems and improve it.

[P3]

#### Training for Human-Robot Collaborative Care

The integration of smart care robots into practice also requires training in human-robot collaboration and related operational support. Participants preferred training and adaptation to be conducted within the facility.

If we are going to learn, I hope it can be done within the facility. That would be more convenient, and it would also help us see whether the robot is suitable for our facility.

[P18]

Clear understanding of service functions and operating procedures was seen as essential.

If we do not even know what it can do or how to use it, it will be like talking to a brick wall, and using it may end up being even more tiring.

[P4]

Hands-on demonstration and direct practice were also considered important for improving mastery.

Training must involve someone teaching us how to operate it by hand, and then we need to explore the details ourselves step by step.

[P29]

In addition, interface clarity and ease of understanding were seen as equally important.

At the very least, both we and the older adults need to know what that button is for. The labels have to be in Chinese. If they are in English, we will not understand them at all.

[P4]

## Discussion

### Principal Findings

This study examined nursing assistants’ expectations of the potential roles of smart care robots, existing facilitating conditions, anticipated barriers, and preparations that may be required before their introduction into long-term care facilities. As most participants lacked direct or sustained experience with care robots, their accounts reflected anticipatory judgments shaped by care experience, news, social media, and everyday observations and therefore require further examination in practice. Participants perceived potential value in safety monitoring, standardized management, repetitive physical tasks, and basic interaction. Openness to new technologies, a supportive organizational climate, and positive external signals provided preliminary favorable conditions, but appeared insufficient to support integration into routine care. Participants anticipated implementation challenges related to unclear human-robot collaboration, limited organizational capacity, potential care-related risks, and insufficient external standards and accountability arrangements. Organizational assessment, contextual adaptation, real-world pilot testing, and human-robot collaboration training were identified as areas warranting attention during preimplementation preparation.

### Anticipated Roles of Smart Care Robots in Long-Term Care

Participants’ expectations of smart care robots extended beyond standardized tasks and repetitive physical work, reflecting the need for continuous observation, timely response, and contextual adjustment in long-term care [48,49]. They expected robots to complement existing care through functions such as dynamic following [50], low-disturbance monitoring [51,52], and immediate response, potentially supporting safety, greater resident autonomy, sleep continuity, and timely care. For emotional support, participants generally considered robots suitable only for basic interaction and entertainment, consistent with previous research suggesting that human care relationships cannot be fully replaced [53]. Notably, the perceived value of robots appeared to lie less in replacing deeper emotional support for residents than in sharing some of nursing assistants’ emotional labor. Long-term care work requires sustained attention, patience, and emotional regulation [54,55], particularly when caring for older adults with cognitive impairment [56]. Participants therefore envisaged robots providing stable, repetitive, and less emotionally complex responses, allowing nursing assistants to focus more on interactions requiring empathy, reassurance, and professional judgment. Previous research has focused mainly on reducing physical workload or improving older adults’ well-being [11,57], with less attention to caregivers’ emotional experiences [58,59]. The current findings therefore suggest that nursing assistants’ emotional labor and work experiences may be useful exploratory dimensions in future real-world evaluations of care robots.

### Initial Openness and Perceived Conditions for Implementation Readiness

This study identified preliminary facilitating conditions at the individual, organizational, and external levels, but these mainly reflected general staff resources, supportive climates, and positive signals rather than concrete implementation capacity. At the individual level, care experience and technological confidence may provide an initial basis for discussing, testing, and adapting robots. More experienced participants were better able to identify potential uses in specific care tasks, whereas those with limited technological experience focused more on learnability, operability, and controllability. Previous research similarly suggests that perceived controllability, transparency, and reliability may influence robot acceptance [60,61]. A small number of younger participants with junior college education appeared to view robots more in terms of workflow integration and human-robot collaboration, broadly consistent with previous findings that age and education may shape technology perceptions [14,62]. However, as this observation was based on only 3 participants (P27, P28, and P30), it should be considered exploratory. Future research could further examine how individual differences relate to implementation readiness and support needs.

A similar gap between openness and implementation capacity was evident at the organizational and external levels. Supportive management and teamwork may facilitate discussion and adaptation [63,64], while social and policy attention to smart eldercare provides a positive context [65]. However, these conditions have not yet translated into specific training, technical support, workflows, or accountability arrangements. Fragmented or exaggerated external information may also make it difficult for participants without direct experience to judge robots’ real-world capabilities. Overall, these facilitating conditions remain limited and may require further development before they can support routine implementation.

### Anticipated Implementation Uncertainties and Perceived Preparatory Needs

Participants anticipated uncertainties concerning organizational capacity, human-robot collaboration, contextual fit, and accountability. Embodied robots generally require continued testing, iteration, and contextual adaptation before stable workflows and responses to unexpected situations can be established [12,66], potentially increasing demands on staffing, time, and technical support in resource-constrained facilities. Human-robot task allocation was a particular concern because, unlike industrial settings with relatively stable task boundaries [67], long-term care requires continuous adjustment to residents’ functional status, cognition, and immediate needs [48,68]. Task allocation must therefore consider care context and the respective capabilities of staff and robots. Moreover, real-world robot use may require staff supervision, technical coordination, and team communication [22,69], potentially creating new work related to coordination and troubleshooting rather than simply reducing workload. Participants consequently questioned how changes in staff responsibilities and workload would be assessed.

Contextual fit and accountability introduced additional uncertainty. Functional limitations, cognitive impairment, and communication difficulties among long-term care residents [70,71] may restrict the applicability of robots designed primarily for users who can clearly express their needs and reliably interact with the technology, potentially raising equity concerns. Responsibility for robot-related incidents may also extend across developers, manufacturers, facilities, and operators [72]. In the absence of adequate standards, reliable information, technical support, and clear accountability arrangements, facilities may bear greater responsibility for managing these risks. Participants therefore identified organizational assessment, rule preparation, contextual adaptation, real-world pilot testing, and human-robot collaboration training as relevant preparatory needs. However, these suggestions reflected anticipatory judgments grounded in care experience and limited technology exposure and require further examination in practice.

### Interpretation in Relation to Existing Frameworks

The findings can be interpreted alongside, rather than as a formal test of, existing technology acceptance and implementation frameworks. Nursing assistants’ accounts of robot usefulness extended beyond efficiency and task support to resident autonomy, timeliness of care, and caregivers’ emotional labor, illustrating how perceptions of usefulness are embedded in care relationships and work contexts. The identified importance of organizational support, external context, human-robot collaboration, and accountability also resonates with the multilevel contextual emphasis of the CFIR [39]. Thus, the analysis provides a context-specific account of individual, organizational, workflow-related, and external conditions surrounding the potential integration of smart care robots from nursing assistants’ perspectives.

### Transferability

Transferability should be considered in relation to the organizational and social context of long-term care facilities in Sichuan Province. Expectations regarding safety monitoring, risk warning, repetitive physical tasks, and basic interaction may reflect broader long-term care needs, including continuous observation, physical workload, and timely responses to residents, which have also been reported internationally [14]. In contrast, concerns about family blame, institutional responsibility, human takeover, and limited transitional support may be more closely tied to local care organization and accountability structures. In this setting, nursing assistants undertake broad direct care responsibilities, while role boundaries and technology-related accountability arrangements remain under development [73]. Similar concerns may therefore take different forms or carry different weight in settings with clearer staffing roles, regulatory standards, and accountability mechanisms. Transferability should accordingly be judged against local staffing, workflows, organizational resources, and governance contexts.

### Limitations

This study was based on interviews from 5 long-term care facilities in Sichuan Province, China. Transferability to other regions and facility types should therefore be considered in relation to local workforce characteristics, organizational resources, and care practices. The sample was predominantly female, older, less educated, and lower in professional rank, which may partly reflect the local nursing assistant workforce but may also limit the range of perspectives represented. Most participants also lacked direct or sustained experience with smart care robots; therefore, their concerns about human-robot collaboration, care risks, and accountability were anticipatory. These views were grounded in care experience but may also have been influenced by news, social media, and everyday observations. Because the sample size was pragmatically determined by operational constraints, the scope of recruitment may have shaped the coverage of the dataset. The breadth and depth of the data varied across analytical categories, with some categories informed by relatively few participants or limited contextual detail. The findings should therefore be interpreted in relation to the scope of the available data and may not encompass the full range of nursing assistants’ perspectives in other settings. Future studies could recruit nursing assistants with more diverse backgrounds from a wider range of facilities to explore perspectives that may be less represented in the current dataset. Future research could also combine real-world pilot testing with longitudinal follow-up to compare preimplementation expectations with actual use experiences and examine how smart care robots are gradually integrated into long-term care practice.

### Conclusions

This study identified nursing assistants’ expected roles for smart care robots and the conditions they considered relevant to their future integration into long-term care practice. Openness to technology, supportive organizational climates, and positive policy and social signals provided an initial foundation, but uncertainties remained around human-robot collaboration, risk management, organizational capacity, and concrete external support. Organizational assessment, contextual adaptation, real-world pilot testing, and practical training may help facilities prepare for implementation, although their feasibility and value require further evaluation.

## Supplementary material

10.2196/97371 Multimedia Appendix 1 Tables supporting the study methods and analysis.

10.2196/97371 Checklist 1 COREQ checklist.

## Abbreviations

CFIR: Consolidated Framework for Implementation Research
COREQ: Consolidated Criteria for Reporting Qualitative Research
TAM: Technology Acceptance Model
UTAUT: Unified Theory of Acceptance and Use of Technology
