# Supplementary material for: Conditions and Pathways for Integrating Smart Care Robots Into Long-Term Care Facilities: Qualitative Study From Nursing Assistants’ Perspectives
Source: J Med Internet Res. 2026 Oct 21;28:e97371. doi: 10.2196/97371 (PMC13686380; doi:10.2196/97371)
Supplement: Supplementary file 2 [file jmir-v28-e97371-s001.docx]

**Multimedia Appendix 2:**

**Supplementary Tables Supporting the Study Methods and Analysis**

**Table S1.** Characteristics of the participating long-term care facilities

| **Facility** | **Operating Model** | **Number of Beds** | **Number of Nursing Assistants** | **Location** |
| --- | --- | --- | --- | --- |
| 1 | Private | 65 | 8 | City centre |
| 2 | Public | 80 | 15 | Suburban area |
| 3 | Private | 150 | 26 | City centre |
| 4 | Publicly built and privately operated | 300 | 50 | Suburban area |
| 5 | Public | 720 | 102 | City centre |

**Table S2.** Illustrative Examples of Initial Coding Across Participants

| **Individual Interviews** | **Initial Coding** |
| --- | --- |
| **P1:** Once, when I was moving a heavier older adult, both of us fell. A robot could probably lift any weight, and the force it used would be more even. | **A1:** Difficulty in transferring heavier older adults;  **A2:** Risk of falls during transfers;  **A3:** Expectation of stable and even robotic lifting |
| **P2:** Some older adults often want to sit up, but sometimes we are too busy and they feel embarrassed to call us. If it were a robot, then whenever they called, it could go and help lift them up straight away. | **A4:** Need for timely care support;  **A5:** Expectation of immediate response to needs;  **A6:** Assistance with sitting-up transfers |
| **P2:** He keeps asking the same questions. No matter what you say, you still have to keep responding, and that can make me feel a bit irritated. A robot has no emotions and can keep giving the same response over and over. | **A7:** Burden of repetitive responses;  **A8:** Emotional exhaustion in repeated communication;  **A9:** Expectation that robots can provide stable repetitive interaction |
| **P3:** Many older adults think they can still walk, but their legs are stiff and their eyesight is poor. If they miss a step, they may fall. It would be good if a robot could remind them or call me for help. | **A10:** Recognition of environmental fall risks;  **A11:** Need for fall warning;  **A12:** Proactive reminder function;  **A13:** Alerting nursing assistants for support |
| **P3:** Sometimes, when we chat with older adults, we cannot know everything. A smart care robot could answer all kinds of questions they ask. | **A14:** Compensation for caregivers’ limited knowledge;  **A15:** Support for diverse communication needs;  **A16:** Expansion of interaction content |
| **P3:** Many older adults have poor eyesight and hearing, so they may not be able to trigger a response from a smart care robot. Not every older adult can use a robot. | **A17:** Sensory limitations of older adults;  **A18:** Barriers to human–robot interaction;  **A19:** Limited applicability of robots |
| **P3:** There needs to be a running-in period to identify its problems and improve it | **A20:** Need for an adaptation period;  **A21:** Problems identified through use;  **A22:** Iterative improvement during use |
| **P4:** It would be good if the robot could follow instructions and move to each bedside, especially to observe and check on agitated older adults. | **A23:** Ward-rounds based on instructions;  **A24:** Monitoring of high-risk residents;  **A25:** Strengthening bedside observation |
| **P4:** If we do not even know what it can do or how to use it, it will be like talking to a brick wall, and using it may end up being even more tiring. | **A26:** Unclear functions and operation may increase burden;  **A27:** Need for clear service scope;  **A28:** Need for clear operating instructions |
| **P4:** At the very least, both we and the older adults need to know what that button is for. The labels have to be in Chinese. If they are in English, we will not understand them at all. | **A29:** Need for a clear and understandable interface;  **A30:** Need for Chinese-language labels;  **A31:** Reducing barriers to use |
| **P5:** Work that requires skill is manageable, but sometimes it is the physical demands that we really cannot keep up with. | **A32:** Heavy physical burden of care;  **A33:** Burden of repetitive physical labour |
| **P6:** If you just press a button and the robot goes to do the task, then what would I be doing? When I am caring for older adults, would I also have to supervise whether it is going to malfunction? | **A34:** Unclear boundaries of human responsibility;  **A35:** Unclear collaborative roles;  **A36:** Concern about supervising robot malfunction |
| **P6:** If something goes wrong, who will the family blame? They will definitely come to the facility and say, ‘We entrusted the older adult to you, and then you handed them over to a robot’. | **A37:** Pressure of family blame;  **A38:** Facility bears ultimate responsibility |
| **P7:** I saw a video online showing a robot hitting an older adult. Is that real or fake? | **A39:** Doubts about the authenticity of external information;  **A40:** Negative information undermines trust |
| **P8:** The adjustment period with newly admitted older adults is when conflicts are most likely to happen. Management worry that family complaints will affect occupancy, so the problem is mainly blamed on the nursing assistant. When I first started this job, an experienced nursing assistant was training me. Once, both of us were fined before I even knew that what we had done was against the rules. | **A41:** High risks during transition periods;  **A42:** Inadequate transmission of rules;  **A43:** Responsibility shifted to frontline staff;  **A44:** Insufficient support during adjustment |
| **P8:** There should be clear operating procedures in place first. If we are left to act based on our own judgement without knowing what is right or wrong, that would be a major concern | **A45:** Need for clear operating procedures;  **A46:** Unclear rules increase concern |
| **P9:** Hygiene involves privacy protection; walking carries the risk of falls; feeding means preventing choking; and older adults can easily become ill when the weather changes. Care work is not as easy as outsiders think, and there are many areas where robots could help us strengthen care. | **A47:** Recognition of care complexity;  **A48:** Recognition of the supportive value of robots;  **A49:** Understanding technology from a professional care perspective |
| **P9:** If I cannot communicate well with an older adult I am responsible for, my colleagues will help and try talking with that person instead. | **A50:** Team mutual support;  **A51:** Peer collaboration;  **A52:** Joint problem-solving in care |
| **P10:** Management needs to investigate first and think it through carefully, to decide whether the robot can be integrated into our team, whether it can work well with everyone, and whether it can help care for older adults properly. | **A53:** Organisational assessment before introduction;  **A54:** Evaluation of team fit;  **A55:** Attention to collaboration and care quality |
| **P10:** If I could see with my own eyes the whole process of it caring for an older adult, and see that it could do it well and skilfully, then I would definitely feel reassured. | **A56:** Need for full-process demonstration;  **A57:** Building trust through actual performance |
| **P11:** When we are too busy, some older adults may not be cared for as carefully as they should be. I feel guilty about that, but there is not much I can do. If there were a smart care robot, it would definitely help. | **A58:** Busy work leads to missed care;  **A59:** Feelings of guilt;  **A60:** Expectation that robots can improve care coverage |
| **P12:** They keep saying that intelligent technology can reduce our care burden, but we still do not know exactly how smart care robots would do that. | **A61:** Gap between publicity and actual practice;  **A62:** Unclear mechanism of workload reduction;  **A63:** Ambiguous perception of technological value |
| **P13:**  With older adults with cognitive impairment, you need to understand their habits and the things that require attention. If communication is difficult, then you should at least respond to their emotions. | **A64:** Adapting to individual habits;  **A65:** Personalised care for cognitive impairment;  **A66:** Need for emotional reassurance |
| **P14:** This is not like a hospital, where doctors and nurses have clearly divided responsibilities. Here, we have to take care of almost everything related to the older adults. What we do each day, and what we do first, often depends on what they need at that moment. By the end of the day, there is almost no real free time. | **A67:** Broad scope of responsibilities;  **A68:** High fluidity of daily tasks;  **A69:** Lack of time to learn and adapt to new technology |
| **P14:** The director really understands our work and is considerate of us. She usually takes the initiative to check in on us, and once during a holiday she gave us 200 yuan to give to our parents. That made up, to some extent, for the guilt we felt inside, and it also made us feel that this work was truly worthwhile. | **A70:** Recognition of labour value;  **A71:** Organisational emotional support;  **A72:** Strengthening professional identity |
| **P15:** At night, we have to check the rooms every two hours. Once older adults are woken up, it is hard for them to fall asleep again. If the robot had something like far-infrared sensing, it could help us check their physical condition without waking them, and it would be more accurate. | **A73:** Need for low-disturbance night monitoring;  **A74:** Maintaining sleep continuity;  **A75:** Monitoring physiological status at night |
| **P16:** Only communication between people carries real emotion. When older adults are in a low mood, they would not want to confide in a robot. The smart care robot can help me with other tasks, and I will talk with the older adult. | **A76:** Deep emotional communication cannot be replaced;  **A77:** Boundary of human–robot role division |
| **P16:** What if the robot does not follow instructions and ends up hurting an older adult? Older adults are not as quick to react as we are, and they may not even be able to get out of the way. | **A78:** Concern about technological loss of control; **A79:** Risk of direct physical harm;  **A80:** Limited ability of older adults to avoid danger |
| **P16:** When we cannot solve a problem, we go to the manager. He is very willing to help and considers everyone’s interests fairly, without favouring anyone. | **A81:** Accessible managerial support;  **A82:** Smooth problem-solving channels;  **A83:** Perceived fairness of management |
| **P16:** When an older adult behaves differently from usual, we need to find out why. For example, if an older adult does not want to eat, a smart care robot should ask further questions about whether they feel physically or emotionally uncomfortable, rather than just leaving. | **A84:** Need to identify the causes of unusual behaviour;  **A85:** Need to explore physical or emotional discomfort;  **A86:** Robots should not end tasks mechanically |
| **P17:** Everyone’s medication is different, and it can change with their condition. When medicines are distributed manually, we are always worried about mistakes. We hope a robot could help sort them out by the right time, the right dose, and for the right person. | **A87:** Complexity of individualised medication management;  **A88:** Concern about medication errors;  **A89:** Right time, right dose, right person |
| **P17:** They say that if robots can share some of the work, we will be able to spend more time with older adults, not that they are meant to replace us. | **A90:** Viewing robots as workload-sharing tools;  **A91:** Workload reduction rather than replacement |
| **P18:** When we turn an older adult, we also need to check whether the diaper needs changing, whether there are any pressure sores, and what the person’s physical condition is like at that moment. Smart care robots should be able to do the same. | **A92:** Multi-task care occurs simultaneously;  **A93:** Need for concurrent condition assessment;  **A94:** Complexity of care situations |
| **P18:** If we are going to learn, I hope it can be done within the facility. That would be more convenient, and it would also help us see whether the robot is suitable for our facility. | **A95:** Preference for in-facility training;  **A96:** Combining learning with real-world observation;  **A97:** Evaluating facility-level fit |
| **P19:** Smart care robots have their own way of operating, and we have human care experience. We can learn from each other and do the job better. | **A98:** Human–robot mutual learning;  **A99:** Collaboration to improve care;  **A100:** Open attitude towards learning |
| **P20:** Some older adults do not sleep at night and keep pressing the call bell. If we are not quick enough, they start shouting and making a fuss, which disturbs other residents’ sleep. Every time, we still have to go over and check on them. | **A101:** Frequent ineffective night calls;  **A102:** Burden of night-time checking;  **A103:** Disturbance to other residents’ sleep |
| **P20:** Some older adults who are still mobile secretly lower the bed rails, or forget to put them back after going to the toilet. That can easily lead to a fall from the bed, so we have to watch for it all the time, and it is hard to guard against. | **A104:** Need to monitor bed-rail status;  **A105:** Prevention of falls from bed;  **A106:** Burden of constant vigilance |
| **P21:** In our facility, we check the expiry dates of the snacks older adults keep in their wardrobes or storage areas every week, but sometimes things are still missed. | **A107:** Need to check food expiry dates;  **A108:** Routine checks may still miss problems |
| **P21:** Machines will definitely malfunction, just like people make mistakes. But for smart care robots used to care for older adults, there should be clear quality standards. You cannot just let any robot enter a facility. | **A109:** Need for specific quality standards;  **A110:** Need for product access thresholds;  **A111:** Awareness of malfunction risks |
| **P21:** In external publicity, they always say that smart care robots do not get tired, do not have emotions, and can keep service quality at a stable standard. | **A112:** Expectation of robotic stability;  **A113:** External publicity shapes perceptions of technological advantages |
| **P22:** Some older adults cry out in pain even when you touch them very lightly. Sometimes they really are uncomfortable, and sometimes they are just in a bad mood and want attention. How would a smart care robot tell the difference? | **A114:** Difficulty in judging real needs;  **A115:** Difficulty recognising emotionally driven expressions;  **A116:** Lack of contextual judgement |
| **P23:** A smart care robot would be like a new colleague, but would the workflow be different? Would I also need to spend time helping it get familiar with the older adults before formal care begins? | **A117:** Unclear human–robot workflow;  **A118:** Unclear way robots fit into care routines;  **A119:** Anticipation of an adaptation period |
| **P23:** You can tell whether a robot is really useful just from one or two details, for example, by asking it to assist with turning an older adult. | **A120:** Need to validate key tasks;  **A121:** Judging usefulness through specific task details |
| **P24:** As soon as you turn away, some older adults try to stand up. It would be good if a smart care robot could stay with them and follow them around, and even chat with them a little. Otherwise, if we keep restraining them all the time, we would feel bad about it. | **A122:** Dynamic following;  **A123:** Real-time risk prevention;  **A124:** Reducing excessive restraint;  **A125:** Balancing safety and freedom |
| **P24:** Semi-dependent older adults need to be transferred many times a day between the bed and the wheelchair. Doing this so often makes our backs hurt. | **A126:** High-frequency transfer tasks;  **A127:** Back strain;  **A128:** Heavy burden of manual handling |
| **P24:** Here, each of us is assigned specific older adults and remains responsible for them until they die. Care is not organised on a rotating basis. Other staff are not very familiar with their situations, so handovers are difficult. Apart from emergencies, people rarely take leave. | **A129:** One-person responsibility system;  **A130:** Concentrated individual responsibility;  **A131:** Difficult handovers;  **A132:** Technology-related risks may fall back on individuals |
| **P25:** When mistakes happen, the facility does not punish us just for the sake of punishment. They talk with us and provide training to help us avoid making the same mistake again. | **A133:** Tolerant management of errors;  **A134:** Improvement-oriented management;  **A135:** Encouraging learning from mistakes |
| **P26:** We have a background in care, so it should be easier for us to learn and understand it. | **A136:** Care background enhances learning confidence;  **A137:** Sense of capability in mastering new technology |
| **P26:** Society is developing, technology is advancing, and there is constant discussion about smart eldercare as the way forward. It feels like eldercare will definitely move in this direction. | **A138:** Recognition of the trend towards smart eldercare;  **A139:** External development signals create positive expectations |
| **P27:** For rehabilitation exercises such as leg lifting, leg stretching, and ankle or wrist movements, smart care robots could completely take over. That would save manpower, and robots could do it more consistently and to a better standard. | **A140:** Standardised rehabilitation tasks can be delegated;  **A141:** Saving manpower;  **A142:** More standardised performance |
| **P27:** Our facility has a monthly sharing meeting where everyone talks about the care problems they have encountered. If smart care robots are introduced in the future, we could definitely discuss them there as well. | **A143:** Mechanism for experience sharing;  **A144:** Platform for collective discussion;  **A145:** Organisational basis for technological adaptation |
| **P27:** It would be better to start with simple supportive tasks that do not involve direct contact with older adults. Not only do we need time to adapt, but older adults also need time. It should be introduced gradually. | **A146:** Starting with low-risk tasks;  **A147:** Gradual trial use;  **A148:** Stepwise transition from indirect to direct care |
| **P27:** Now many robots are equipped with radar and similar functions, which can provide real-time feedback on residents’ conditions so that I can respond in time | **A177: Recognition of real-time monitoring functions A178: Expectation of timely feedback on residents’ conditions A179: Improving care responsiveness through technological support** |
| **P28:** Some older adults have hypertension. When they stand up, their blood pressure may change, which could cause them to fall. | **A149:** Need to identify physiological abnormalities;  **A150:** Monitoring postural change risks;  **A151:** Fall warning linked to physiological changes |
| **P28:** Robots have machine learning and adaptive capabilities, and I can input residents’ care preferences so that they can learn them | **A180: Recognition of robot learning and adaptive capabilities A181: Expectation of personalised care through adaptive technology A182: Viewing robots as collaborative partners in care delivery** |
| **P28:** Tasks like telling stories, playing music, having simple conversations, or answering questions about the weather are completely manageable for a robot | **A152:** Robots can undertake basic interaction;  **A153:** Entertainment and companionship functions;  **A154:** Support for simple communication |
| **P29:** Right now, I care for seven or eight older adults. If robots are introduced, they will definitely ask me to care for more people. So has my workload actually changed, and how would my pay change? | **A155:** Technology may increase workload rather than reduce it;  **A156:** Unclear changes in workload;  **A157:** Uncertain remuneration and return |
| **P29:** He wants to stand up, but says ‘sit down’ instead. Could the robot understand that? | **A158:** Inconsistency between speech and intention;  **A159:** Risk in interpreting verbal commands;  **A160:** Difficulty in judging intended action |
| **P29:** If a robot is helping an older adult put on trousers, it cannot judge whether the person’s leg can bend or lift, or whether this might cause a fracture or injury. It would just follow the instruction rigidly. | **A161:** Lack of individualised care judgement;  **A162:** Rigid task execution;  **A163:** Risk of injury during operation |
| **P29:** Training must involve someone teaching us how to operate it by hand, and then we need to explore the details ourselves step by step. | **A164:** Need for hands-on training;  **A165:** Demonstration-based teaching;  **A166:** Learning through gradual exploration |
| **P30:** Every day, we arrange for older adults to follow exercise videos, but some just sit there and do not want to move. A smart care robot could go over to them like we do, encourage them, and guide them through the exercises. That would be better than just using a television. | **A167:** Increasing older adults’ engagement;  **A168:** Active prompting of activities;  **A169:** Embodied interaction is superior to passive media |
| **P30:** Our nursing staff here are mostly older; I would definitely be the main operator. If the institution introduces robots, I would be the first to learn how to use them and then teach the older colleagues | **A173: Willingness to become an early technology adopter; A174: Confidence in learning smart care robot operation; A175: Peer teaching and knowledge sharing among nursing assistants; A176: Facilitating technology adoption through internal support.** |
| **P31:** If it detects that an older adult is in danger but does not issue an alarm, is that the nursing assistant’s fault for not checking in time, or the smart care robot’s fault? The robot itself cannot take responsibility. | **A170:** Unclear attribution of responsibility;  **A171:** Accountability dilemma after alarm failure;  **A172:** Robots cannot bear responsibility |

**Table S3.** Representative Examples of Analytical Framework Development

| **Raw data extract** | **Initial codes** | **Working analytical category** | **Final analytical category** | **Final analytical domain** |
| --- | --- | --- | --- | --- |
| **P1:** “Once, when I was moving a heavier older adult, both of us fell. A robot could probably lift any weight, and the force it used would be more even.” | Difficulty in transferring heavier older adults; risk of falls during transfers; expectation of stable and even robotic lifting | Physical workload and transfer assistance | Support for Repetitive Physical Tasks | Real-World Care Needs |
| **P3:** “Many older adults think they can still walk, but their legs are stiff and their eyesight is poor. If they miss a step, they may fall. It would be good if a robot could remind them or call me for help.” | Recognition of environmental fall risks; need for fall warning; proactive reminder function; alerting nursing assistants for support | Risk detection and timely warning | Support for Safety Monitoring and Risk Warning | Real-World Care Needs |
| **P2:** “He keeps asking the same questions… A robot has no emotions and can keep giving the same response over and over.” | Burden of repetitive responses; emotional exhaustion in repeated communication; expectation that robots can provide stable repetitive interaction | Repetitive interaction support | Basic Interaction and Emotional Support | Real-World Care Needs |
| **P14:** “The director really understands our work and is considerate of us. She usually takes the initiative to check in on us…” | Recognition of labour value; organisational emotional support; strengthening professional identity | Supportive management environment | A Supportive Organisational Climate and Inclusive Management Practices | Limited Facilitating Conditions |
| **P26:** “We have a background in care, so it should be easier for us to learn and understand it.” | Care background enhances learning confidence; sense of capability in mastering new technology | Confidence in technological learning | Nursing Assistants’ Openness to Technological Change | Limited Facilitating Conditions |
| **P6:** “When I am caring for older adults, would I also have to supervise whether it is going to malfunction?” | Unclear boundaries of human responsibility; unclear collaborative roles; concern about supervising robot malfunction | Role, supervision, and responsibility uncertainty | Unclear Models of Human–Robot Collaborative Care | Major Anticipated Barriers |
| **P22:** “Sometimes they really are uncomfortable, and sometimes they are just in a bad mood and want attention. How would a smart care robot tell the difference?” | Difficulty in judging real needs; difficulty recognising emotionally driven expressions; lack of contextual judgement | Difficulty interpreting context-dependent care needs | Potential New Care Risks Introduced by Technology Use | Major Anticipated Barriers |
| **P21:** “For smart care robots used to care for older adults, there should be clear quality standards. You cannot just let any robot enter a facility.” | Need for specific quality standards; need for product access thresholds; awareness of malfunction risks | External quality assurance and access requirements | Weak External Support Conditions | Major Anticipated Barriers |
| **P10:** “Management needs to investigate first and think it through carefully, to decide whether the robot can be integrated into our team…” | Organisational assessment before introduction; evaluation of team fit; attention to collaboration and care quality | Pre-implementation organisational assessment | Organisational Assessment and Rule Preparation | Preparatory Pathways for Implementation |
| **P18:** “When we turn an older adult, we also need to check whether the diaper needs changing, whether there are any pressure sores, and what the person’s physical condition is like…” | Multi-task care occurs simultaneously; need for concurrent condition assessment; complexity of care situations | Adaptation to concurrent and changing care needs | Improving Contextual Adaptability | Preparatory Pathways for Implementation |
| **P23:** “You can tell whether a robot is really useful just from one or two details, for example, by asking it to assist with turning an older adult.” | Need to validate key tasks; judging usefulness through specific task details | Validation through real-world care tasks | Pilot Application in Real-World Care Settings | Preparatory Pathways for Implementation |
| **P29:** “Training must involve someone teaching us how to operate it by hand, and then we need to explore the details ourselves step by step.” | Need for hands-on training; demonstration-based teaching; learning through gradual exploration | Practical and experiential training | Training for Human–Robot Collaborative Care | Preparatory Pathways for Implementation |

**Note.** The analysis followed a combined deductive–inductive orientation. The research questions and interview guide provided 3 a priori overarching analytical domains: expected care functions, conditions influencing integration into routine care, and preparations required before implementation. Facilitating and hindering factors, organisational support, training, and external support were acknowledged as a priori areas of interest. Within and beyond this initial structure, the specific codes, working categories, category definitions, and boundaries were developed and refined through comparison within and across participants’ accounts. During this process, the initial domain concerning conditions influencing integration was divided into limited facilitating conditions and major anticipated barriers. TAM, UTAUT, and CFIR served as sensitising concepts but were not used to map or rate the data systematically against their predefined constructs.

**Table S4.** Illustrative Excerpt of Charted Participant Data Across Selected Analytical Categories

| **Participant** | **Safety Monitoring and Risk Warning** | **Nursing Assistants’ Openness to Technological Change** | **A Supportive Organisational Climate and Inclusive Management Practices** | **Unclear Models of Human–Robot Collaborative Care** | **Potential New Care Risks Introduced by Technology Use** | **Pilot Application in Real-World Care Settings** |
| --- | --- | --- | --- | --- | --- | --- |
| P4 | Robots were expected to follow instructions, move between bedsides, and strengthen observation of agitated or otherwise high-risk residents. | — | — | — | — | — |
| P6 | — | — | — | Expressed uncertainty about whether nursing assistants would need to supervise robot performance and where human–robot roles and responsibilities should lie. | — | — |
| P16 | — | — | Accessible and fair managerial support was described as facilitating problem-solving in daily care. | — | Concerned that failure to follow instructions appropriately could cause direct physical harm to vulnerable older adults. | — |
| P19 | — | Human care experience and robot operating capabilities were viewed as complementary, supporting mutual learning and collaboration. | — | — | — | — |
| P23 | — | — | — | Expressed uncertainty about how robots would alter existing care workflows and whether nursing assistants would need to help robots adapt to residents before formal care began. | — | Suggested testing specific care tasks, such as assisting with turning, to judge practical usefulness before broader deployment. |
| P29 | — | — | — | Expressed concern that robot introduction could lead nursing assistants to care for more residents, creating uncertainty about workload redistribution and remuneration. | Concerned that robots might execute instructions rigidly without adequately considering individual physical limitations, potentially causing injury. | — |

**Note.** This table presents an illustrative excerpt from the full framework matrix. Participants are represented in rows and selected analytical categories in columns. Cells contain condensed summaries of participants’ accounts rather than verbatim quotations. An em dash indicates that no substantive data were charted for that participant under the selected analytical category in the full framework matrix. Only selected participants and analytical categories are shown for transparency and brevity.

**Table S5.** Final Analytical Framework

| **Analytical domain** | **Analytical category** | **Definition and scope** |
| --- | --- | --- |
| **Real-World Care Needs** | Support for Safety Monitoring and Risk Warning | Expectations that smart care robots support continuous observation, identification of risks or abnormalities, timely reminders, alerts, and responses to safety-related events. |
|  | Support for Standardised Checking and Management | Expectations that robots assist with routine, repetitive, and standardised checking or management tasks in daily care. |
|  | Support for Repetitive Physical Tasks | Expectations that robots undertake repetitive, physically demanding, or standardisable care activities to reduce physical workload. |
|  | Basic Interaction and Emotional Support | Expectations that robots provide simple conversation, repetitive responses, entertainment, companionship, and other basic forms of interaction while recognising the continued importance of human emotional care. |
| **Limited Facilitating Conditions** | Nursing Assistants’ Openness to Technological Change | Nursing assistants’ willingness, confidence, and perceived ability to learn about and work with smart care robots. |
|  | A Supportive Organisational Climate and Inclusive Management Practices | Existing managerial support, fairness, teamwork, tolerance of errors, and opportunities for collective discussion that may facilitate adaptation to new technology. |
|  | External Recognition of the Value and Potential of Intelligent Care | Positive social, policy, and informational signals that reinforce expectations regarding the potential value of smart eldercare technologies. |
| **Major Anticipated Barriers** | Inadequate Organisational Resources and Implementation Conditions | Constraints related to staffing, time, workload, organisational capacity, and other resources required to introduce and sustain robot use. |
|  | Unclear Models of Human–Robot Collaborative Care | Uncertainty regarding role allocation, task boundaries, supervision, workflow integration, workload redistribution, and responsibility between staff and robots. |
|  | Potential New Care Risks Introduced by Technology Use | Concerns that robots may misinterpret care needs, fail to adapt to individual conditions, malfunction, or introduce new physical and care-related risks. |
|  | Weak External Support Conditions | Insufficient external standards, product access requirements, reliable information, technical support, and accountability arrangements for smart care robots. |
| **Preparatory Pathways for Implementation** | Organisational Assessment and Rule Preparation | Need to assess organisational and team fit and establish operating procedures, responsibility arrangements, and rules before formal introduction. |
|  | Improving Contextual Adaptability | Need to improve robots’ ability to recognise individual differences, changing conditions, multiple concurrent care needs, and context-dependent expressions or behaviours. |
|  | Pilot Application in Real-World Care Settings | Need for staged testing in actual care situations to evaluate key tasks, identify problems, build trust, and support iterative refinement. |
|  | Training for Human–Robot Collaborative Care | Need for practical, hands-on, context-specific training, demonstrations, gradual learning, and peer or organisational support for robot use. |

**Note.** The final analytical framework combined a priori domains informed by the research questions and interview guide with categories developed and refined through participants’ accounts. The initial domain concerning conditions influencing integration was divided during analysis into limited facilitating conditions and major anticipated barriers. TAM, UTAUT, and CFIR were used as sensitising concepts rather than as comprehensive coding templates.

**Table S6.** Summary of Member Checking Procedures and Outcomes

| **Participant ID** | **Completion method** | **Time From Initial Contact to Completion** | **Summary of participant feedback** |
| --- | --- | --- | --- |
| **P1** | Face-to-face follow-up | Day 8 | Confirmed that the summary was consistent with their views and provided no additional comments. |
| **P2** | Face-to-face follow-up | Day 8 | Confirmed that the summary was consistent with their views and reiterated that robots could assist with some repetitive care tasks and simple interactions. |
| **P3** | Face-to-face follow-up | Day 8 | Confirmed that the summary was consistent with their views and reiterated the importance of safety monitoring and the potential risks that may arise during robot use. |
| **P4** | Face-to-face follow-up | Day 9 | Confirmed that the summary was consistent with their views and provided no additional comments. |
| **P5** | Face-to-face follow-up | Day 9 | Confirmed that the summary was consistent with their views and provided no additional comments. |
| **P6** | Face-to-face follow-up | Day 9 | Confirmed that the summary was consistent with their views and reiterated the need to clarify responsibility if problems occur after robots become involved in care. |
| **P7** | Face-to-face follow-up | Day 9 | Confirmed that the summary was consistent with their views and provided no additional comments. |
| **P8** | **WeChat** | Same day | Confirmed that the summary was consistent with their views and provided no additional comments. |
| **P9** | **WeChat** | Day 4 | Confirmed that the summary was consistent with their views and provided no additional comments. |
| **P10** | **WeChat** | Same day | Confirmed that the summary was consistent with their views and provided no additional comments. |
| **P11** | **WeChat** | Day 2 | Confirmed that the summary was consistent with their views and provided no additional comments. |
| **P12** | Face-to-face follow-up | Day 9 | Confirmed that the summary was consistent with their views and provided no additional comments. |
| **P13** | Face-to-face follow-up | Day 9 | Confirmed that the summary was consistent with their views and provided no additional comments. |
| **P14** | Face-to-face follow-up | Day 9 | Confirmed that the summary was consistent with their views and provided no additional comments. |
| **P15** | Face-to-face follow-up | Day 9 | Confirmed that the summary was consistent with their views and reiterated that nighttime monitoring should minimise disruption to older adults’ sleep. |
| **P16** | Face-to-face follow-up | Day 10 | Confirmed that the summary was consistent with their views and reiterated that robots cannot replace genuine emotional communication between people. |
| **P17** | Face-to-face follow-up | Day 10 | Confirmed that the summary was consistent with their views and provided no additional comments. |
| **P18** | Face-to-face follow-up | Day 10 | Confirmed that the summary was consistent with their views and reiterated the need for training before robots are used in practice. |
| **P19** | Face-to-face follow-up | Day 10 | Confirmed that the summary was consistent with their views and provided no additional comments. |
| **P20** | Face-to-face follow-up | Day 10 | Confirmed that the summary was consistent with their views and reiterated the importance of nighttime safety monitoring and timely responses. |
| **P21** | Face-to-face follow-up | Day 10 | Confirmed that the summary was consistent with their views and reiterated that care robots should be subject to clear quality and use standards. |
| **P22** | **WeChat** | Day 4 | Confirmed that the summary was consistent with their views and reiterated that robots may have difficulty identifying some of the more complex needs of older adults. |
| **P23** | **WeChat** | Day 4 | Confirmed that the summary was consistent with their views and provided no additional comments. |
| **P24** | **WeChat** | Day 2 | Confirmed that the summary was consistent with their views and provided no additional comments. |
| **P25** | **WeChat** | Day 2 | Confirmed that the summary was consistent with their views and provided no additional comments. |
| **P26** | **WeChat** | Day 2 | Confirmed that the summary was consistent with their views and reiterated that robots would be more appropriate as an assistive tool rather than as a complete replacement for human care. |
| **P27** | **WeChat** | Same day | Confirmed that the summary was consistent with their views and reiterated that robots could be introduced gradually and that staff would also require relevant training. |
| **P28** | **WeChat** | Same day | Confirmed that the summary was consistent with their views and reiterated that robots need to better adapt to the characteristics and care needs of different older adults. |
| **P29** | **WeChat** | Day 4 | Confirmed that the summary was consistent with their views and reiterated concerns about workload, responsibility, and operational training following the introduction of robots. |
| **P30** | **WeChat** | Same day | Confirmed that the summary was consistent with their views and provided no additional comments. |
| **P31** | **WeChat** | Day 2 | Confirmed that the summary was consistent with their views and provided no additional comments. |
